# Spectroscopic Techniques in Bacterial Analysis: Applications of FTIR and Raman—Review

**DOI:** 10.3390/foods15040644

**Published:** 2026-02-11

**Authors:** Elisa Audin, Panagiota Dima, Ioannis S. Chronakis, Ana C. Mendes

**Affiliations:** Research Group for Food Production Engineering, Technical University of Denmark (DTU)-Food, Henrik Dams Allé B202, 2800 Kgs. Lyngby, Denmark

**Keywords:** probiotics, spectroscopy, biomolecules, infrared, Raman, data analysis

## Abstract

The growing recognition of probiotics’ beneficial effects on human health has significantly increased the need to identify, quantify, and characterize these microorganisms. In this context, Fourier-transform infrared (FTIR) and Raman spectroscopies have become indispensable analytical tools in probiotic research, offering non-invasive, rapid, and precise insights into the molecular structure and composition of probiotic strains. Likewise, these spectroscopic methods have also been shown relevant to investigate other bacterial species beyond probiotics. This review explores the principles of FTIR and Raman spectroscopies, emphasizing their role in identifying key biomolecules within bacterial cells, with particular focus on probiotics. Key applications of these vibrational spectroscopies in bacterial research include analyzing cell composition, evaluating encapsulation techniques, and monitoring responses to environmental stress, all of which contribute to enhanced stability and efficacy of probiotic formulations. Furthermore, FTIR and Raman spectroscopies assist in strain identification, investigation of bacteria-water interactions, and quality control, thereby supporting improved formulation and quality assurance. Collectively, these techniques demonstrate significant potential to drive innovation in the probiotics industry through precise strain customization, improved product stability, and robust quality control processes.

## 1. Introduction

Probiotics are live microorganisms that, when consumed in adequate amounts, offer health benefits to the host [1], including inhibiting pathogens, aiding digestion, enhancing nutrient absorption, and modulating gut microbiota [2]. Predominantly from the genera *Lactobacillus* and *Bifidobacterium*, many probiotic strains naturally inhabit the human gut, with their presence influenced by environmental, biological, and genetic factors [3,4,5]. Regular intake of probiotics through food or supplements supports gut flora development and immune function [6], and has shown beneficial effects in managing conditions such as inflammatory bowel disease [7,8], inflammatory and irritable rheumatoid arthritis [9], colorectal cancer [8], cardiovascular [10], atopic dermatitis, lactose intolerance, gastroenteritis, and food allergy [9].

Characterization methods based on vibrational spectroscopy have advanced significantly and are now widely applied to the analysis of probiotics and bacteria in general [11,12,13]. Those methods can be used both for qualitative and quantitative analysis and offer the advantage of being non-destructive, fast, inexpensive, and solvent-free, and allow real-time and in situ analysis [13]. Furthermore, these methods are easy to use, require little or no sample preparation, and can analyze both organic and inorganic matrices, either solid, liquid, or gaseous [14]. These spectroscopic methods are reproducible and can provide analysis of several matrix components in a single spectrum [15].

Therefore, FTIR and Raman spectroscopies can be used in several industries, and are used in the food industry, mainly for raw material analysis, product quality control, and process management [16,17,18,19]. In the context of probiotic research, those spectroscopies allow the analysis of the main components of probiotics, and in particular, the probiotics’ membrane components, and thus provide relevant information to identify probiotic strains, isolated or present in food samples, for example.

This paper offers a comprehensive review of recent advances in the application of Fourier-transform infrared (FTIR) and Raman vibrational spectroscopies for the study of bacterial microorganisms, with a particular emphasis on probiotics. Specifically, it explores how these cutting-edge vibrational methods can be employed to assess key aspects of probiotics, such as their taxonomy, quantification, and viability. The objective is to demonstrate the versatility and potential of FTIR and Raman spectroscopies in probiotic research, offering insights into their mechanisms of operation and how they can be applied across various fields.

Additionally, the paper delves into a wide range of information that can be obtained from these technologies, from detailed microbial fingerprinting to real-time viability assessments. It also provides an in-depth overview of the sample preparation techniques required for optimal use of these spectroscopic methods, as well as a discussion of their strengths, limitations, and future potential in advancing probiotic bacteria research.

## 2. FTIR and Raman Fundamentals

These methods are based on the following basic principle: the irradiated sample interacts with light, which excites its molecules, causing vibrational transitions and motions [20]. Depending on the technology used, the mechanism of light absorption (FTIR) or scattering (Raman) will be recorded by the spectrometer and will provide bands assigned to specific vibrational movements of the molecules [15]. For both methods, measurements are performed in the infrared region of the electromagnetic spectrum. This infrared (IR) range includes several regions: the near-infrared (NIR) (750–2500 nm), the mid-infrared (MIR) (2500–40,000 nm), and the far-infrared (40,000–60,000 nm) [13]. Several compounds, both organic and inorganic, have “spectral signatures” in the infrared range, linked to molecular vibrations. By exploring the vibrational motions of the molecules present in the sample (e.g., bacteria, food systems), these methods provide information on their structure, the chemical bonds of their component molecules, and more generally on the chemical composition of the sample [21,22]. IR and Raman spectroscopies provide complementary information since some vibrational modes of molecules are active in FTIR and others are active in Raman, allowing a complete analysis of the structure and composition of samples. Functional groups (composing the molecules of the sample) that are very sensitive to dipole moments react well to IR technology. On the other hand, functional groups that are not very sensitive to dipole moments but more to polarizability respond better to the Raman method [23].

In IR spectroscopy, the sample is irradiated with infrared light, exciting molecular vibrations and causing absorption at characteristic frequencies [15]. Molecules at room temperature are in their fundamental vibrational state; upon absorbing a quantum of light, they transition to higher vibrational states, producing a spectrum from these energy changes [22]. Absorption bands depend on bond strength and atomic mass, with positions expressed in wavenumbers (cm^−1^), directly proportional to absorbed energy [24].

**Figure 1:**
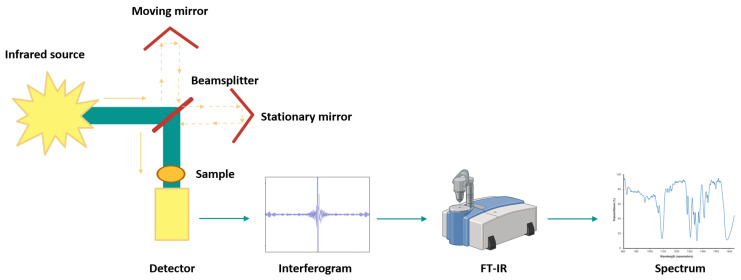
Simplified optical layout of a typical FTIR spectrometer (Created with BioRender.com).

FTIR, commonly used in the mid-infrared range, employs Fourier transformation to improve signal stability and reduce noise [22,25]. The system (Figure 1) consists of a radiation source, an interferometer, and a detector. IR radiation passes through the interferometer, where a beam splitter directs light to fixed and moving mirrors. The recombined beam interacts with the sample, and the resulting interferogram is converted into a spectrum using Fourier transform algorithms [22].

This method can be applied to the surface of a sample (e.g., bacteria, material, ingredient, and or food), and the quality of the spectrum depends mainly on the homogeneity of the sample at the time of measurement, the particle size, and thickness. For qualitative or quantitative analysis in bacteria research, it is important, before measurement, to concentrate on the bacteria and remove water effectively [24,26]. Using FTIR spectroscopy for aqueous samples could cause unwanted interference due to the strong absorption of water in the MIR range, and requires laborious sample preparation [22]. However, different types of FTIR can be used to overcome such challenges, which include Transmission [27], Specular Reflectance [28,29], Reflection Absorption [28,29], Diffuse Reflectance (DRIFTS) [29,30], and Attenuated Total Reflectance (ATR) [31]. ATR is the most familiar one due to its simplicity and the wide range of testing materials, including aqueous samples. This technique requires little or no sample preparation and allows the study of aqueous samples, including wet bacteria, by subtracting the spectral contribution of water from the spectrum obtained [32,33].

NIR relies on the absorption of light by overtone and combination bands of molecular vibrations, with penetration depth varying across the short- and long-wavelength NIR regions. It offers advantages such as deeper penetration and the absence of sample dilution requirements; however, spectral interpretation is challenging, and light scattering is significant [34,35]. NIR grating spectrometers use diffraction gratings to separate light into its component wavelengths, measuring each wavelength sequentially, and, in general, provide higher spectral resolution and detailed analysis than NIR due to precise wavelength separation. However, the resolution of both NIR and NIR grating spectrometers is generally lower compared to FTIR, which can be a limitation for detailed molecular analysis. FTIR provides high sensitivity and detailed spectral information, suitable for complex sample analysis and for the identification of specific molecular structures. MIR relies on the absorption of radiation by molecular motions, giving better specificity and reproducibility, being easier in spectral interpretation, nonetheless requiring more sample preparation, and having interference from water [36].

**Figure 2:**
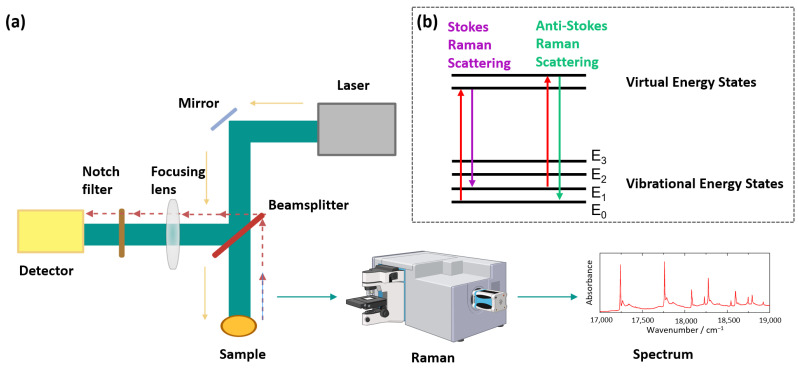
(**a**) Simplified optical layout of a typical Raman spectrometer, and (**b**) the schematic energy diagrams of electronic and vibrational transitions in Raman scattering (created with BioRender.com).

Raman spectroscopy analyzes inelastic scattering of monochromatic light (visible or IR laser, 4000–400 cm^−1^), as shown in Figure 2, rather than absorption as in the IR spectroscopy matrix [20], having minimum interference from water, providing easy analysis of materials, and delivering direct chemical information [37,38]. Most scattering is elastic (Rayleigh), but about 1 in 10^6^–10^8^ photons undergo Raman scattering, producing frequency shifts due to energy exchange between photons and molecules [39]. These shifts, known as the Raman effect, generate Stokes and anti-Stokes lines, which reveal the vibrational information of molecules [14,40]. The scattered light is filtered, dispersed by a diffraction grating, and detected to produce the Raman spectrum [39].

There are different variants of Raman spectroscopy with more advanced systems to improve the quality of the measurement, and particularly, the sensitivity of the method, which is initially low [25]. Spontaneous Raman spectroscopy [41], tip-enhanced Raman spectroscopy (TERS) [42], coherent anti-Stokes Raman spectroscopy (CARS) [43], surface-enhanced Raman spectroscopy (SERS) [44,45], and Fourier transform Raman spectroscopy (FTRS) [46] are the most commonly used systems [43,44,45,46,47,48].

## 3. Methods for Bacterial Sample Preparation and Analysis for Raman and FTIR Spectroscopies

### 3.1. Sample Preparation

For bacterial sample analysis through Raman and FTIR, the composition of the sample and its cell concentration are two of the most important parameters that need to be considered. Bacterial sample preparation is often unnecessary for vibrational spectroscopy. For instance, probiotics in dry powder form can be analyzed directly using FTIR or Raman without preparation [20]. Solid samples, like freeze-dried bacterial pellets, can also be analyzed directly. For FTIR of probiotic powders, potassium bromide (KBr) is commonly used. Freeze-dried lactic bacteria were similarly mixed with KBr, pressed, and stored with silica gel before analysis [47]. Encapsulated probiotics can also be directly analyzed using FTIR with an ATR accessory [48,49].

However, certain types of analysis require sample preparation. The most used preparation methods for the analysis of bacteria, and more especially, probiotics, are cultivation and filtration.

Cell cultivation is the simplest method for preparing bacterial samples and provides a large amount of biomass for analysis. For both FTIR and Raman spectroscopies, bacteria can be grown either on solid or liquid media for sample preparation [50]. The sample is spread on FTIR/Raman windows (e.g., zinc selenide [51], calcium fluoride [52]) and dried under moderate heat or in a desiccator to form a transparent film. Other protocols have been described elsewhere [53,54,55,56,57].

For liquid sample preparation, cells are typically concentrated using membrane filter analysis [58] or centrifugation [59,60]. Anodic membranes are preferred for FTIR measurements as they do not interfere with the 1400–1000 cm^−1^ range. Dilution may be necessary to prevent high cell density, which complicates analysis. After filtration, membranes are washed with distilled water, air-dried for 60 min, and then analyzed using ATR-FTIR [61]. For Raman analysis, high-purity alumina or silver membranes are commonly used [54].

### 3.2. Spectral Analysis

Spectral analysis from vibrational spectroscopies (e.g., FTIR and Raman) involves assigning spectral bands to the chemical components of the sample. The IR spectra of bacterial cells allow not only to identify functional groups, but also to detect changes in the molecular structure, such as interactions between components (e.g., hydrogen bonds, lipid-protein interactions) and conformational states (e.g., protein structures) [62], mostly at the bacterial membrane. It is important to note that environmental factors can shift band appearances, particularly those related to hydrogen bonding [13]; thus, standardizing sample preparation is crucial to prevent variations in the spectra.

The various studies that have been carried out using FTIR and Raman spectroscopies for the study of biological matrices have allowed the collection of band assignments characteristic of these biological samples. Table 1 provides a summary of the main band assignments identified for biological samples found by these two spectroscopic methods [63,64]. Several spectral ranges could be associated with certain functional groups and thus with certain characteristic molecules: 3000–2800 cm^−1^ associated with the fatty acids present in bacteria, 1700–1500 cm^−1^ associated with the Amide I and amide II bands of proteins and peptides, 1500–2000 cm^−1^ associated with both the bending vibrations of fatty acids, proteins and phosphate bearing compounds, 1200–900 cm^−1^ associated with the absorption band of membrane polysaccharides, and 900–700 cm^−1^ considered as the “fingerprint” region, which gathers low absorbance values, specific to some bacteria [65].

### 3.3. Data Processing and Statistical Analysis

Spectrum analysis typically involves three steps: pre-processing, model building, and application, common to both FTIR and Raman spectroscopy [25]. Pre-processing improves spectrum quality by reducing noise and correcting interference using techniques such as SNV, MSC, smoothing (Savitzky–Golay), baseline correction, and derivatives [19,53,56,66,67,68,69]. Widely used methods include vector normalization and extended multiplicative signal correction for accurate quantification [70].

After pre-processing, chemometric methods convert spectral data into essential information by reducing multidimensional data to principal components for analyte separation and calibration sample [14,26]. Depending on prior knowledge, methods can be unsupervised (e.g., PCA, HCA, FA) or supervised, with unsupervised approaches often applied in food microbiology for species discrimination [71].

Supervised chemometrics require prior knowledge (e.g., cell density and nucleic acid sequence) and are used for qualitative identification or quantitative analysis, such as CFU determination [26]. Common methods include LDA, PLSR, and SIMCA for classification, and algorithms like MLR, PLS, and PCR for quantitative modeling of samples [13,26,71]. Calibration models must use representative data and be validated with independent samples [13].

Model performance assesses quality through cross-validation and external validation using metrics such as RMSEC, RMSECV, RMSEP, and R^2^ values [25]. However, to facilitate a more robust comparison between models and previously reported studies, normalized performance indices such as the residual predictive deviation (RPD) and the range error ratio (RER) are also considered. Reliable and robust models are characterized by high R^2^ values, low RMSE values, minimal differences between calibration and validation errors, and sufficiently high RPD and RER values, which indicate strong predictive accuracy relative to data variability and range [15]. For classification analysis, accuracy, sensitivity, specificity, and Cohen’s kappa are calculated from confusion matrices [68,72].

For bacterial identification using FTIR or Raman, it is essential to build spectroscopic databases under controlled conditions (growth medium, temperature, stage). First, spectra from single bacteria with sufficient replicates are analyzed to create a classification model. Validation requires an independent dataset, and once validated, a third dataset can be used for identifying isolated samples from environmental or clinical sources [58,73].

### 3.4. Artificial Intelligence in Spectroscopic Analysis

In recent years, Artificial Intelligence (AI) and machine learning (ML) have emerged as transformative tools in various scientific disciplines, including spectroscopy [74]. The integration of AI and ML algorithms with FTIR and Raman spectroscopy is revolutionizing bacterial analysis by enhancing data processing capabilities and expanding the range of applications [75]. AI offers powerful tools for automating complex analysis tasks, extracting relevant features from spectral data, classifying bacteria with high accuracy, and making predictions about their properties and behaviors [76].

As already mentioned, several critical preprocessing steps are commonly employed to enhance the quality and accuracy of FTIR and Raman spectral data obtained from bacterial samples. Emerging AI-assisted methods, such as convolutional neural networks (CNNs) and autoencoders, offer the potential for automated and more accurate baseline correction by learning complex baseline patterns from large datasets [77,78]. Noise reduction could also be facilitated by deep learning models like autoencoding neural networks and CNNs, which have shown promise in effectively removing instrumental noise and spectral artifacts by learning complex noise patterns from training data [79]. The normalization and spectral alignment that are essential for reducing variability in spectral data could be performed by spectral alignment algorithms, correcting minor shifts in wavenumber that can occur due to instrumental variations or sample conditions, ensuring that corresponding spectral features are properly aligned across different spectra [80]. Moreover, advanced methods leveraging chemometrics or AI algorithms are also being explored to disentangle complex spectral mixtures and isolate the signals of interest [75].

## 4. Applications of FTIR and Raman in Bacteria Analysis

FTIR and Raman spectroscopies have been mainly used for taxonomy, biochemical, and biophysical evaluation of bacteria, including probiotics [13]. They allow the study of a wide range of biochemical properties of bacteria and particularly of their membrane components. They can be used to identify bacteria (at the species or strain level), quantify them, and study their viability and response or state after stress [26]. Furthermore, these vibrational spectroscopic techniques have also been used to investigate the interactions between probiotics and excipients or coating materials, the encapsulation, and drying processes. In food quality and safety applications, FTIR, NIR, and Raman spectroscopy combined with multivariate analysis offer rapid, high-throughput, and cost-effective metabolomic fingerprinting. These methods allow the detection of key food constituents and the identification of bacteria, fungi, and yeasts in complex food matrices [81,82]. Furthermore, FTIR-Raman systems enable real-time, non-invasive monitoring of microbial activities (e.g., fermentations) by tracking metabolic products and pH changes without sampling [63,83]; FTIR-ATR and Raman spectroscopy have also been used to characterize biofilms formed by food-related bacteria, supporting strain differentiation relevant to food spoilage and safety [63,84]. In this review, the information is organized into sections based on the following applications: taxonomic analysis and bacterial identification, quantification of bacterial viability, assessment of bacterial stability, and monitoring of bioprocesses, as described below. Table 2 summarizes some types of applications where IR and Raman vibrational spectroscopy methods have been used.

### 4.1. Taxonomic Analysis and Bacterial Identification

FTIR and Raman spectra provide “phenotypic fingerprints” of the bacteria, which can then be used for taxonomic analysis and identification of microorganisms according to their species or strain [13]. FTIR spectroscopy was used to identify species of Bifidobacterium strains isolated from food and human feces. The FTIR spectra of each unknown microorganism were compared with a library of existing strain spectra (obtained by the 16S rDNA method). Food isolates showed similarities to the type strains of *B. animalis*, *B. lactis* and *B. longum*. The identification of Bifidobacteria from feces using FTIR spectra was not conclusive; thus, subsequent fermentation tests and 16S rDNA sequencing allowed further characterization of the different under-study isolates [50]. Prabhakar and Harper were able to classify several microorganisms, including probiotics, down to the strain level (*S. thermophillus*, *Propionibacterium freudenreichii* and *Lactobacillus* spp.) by combining the use of hydrophobic grid membrane filters and FTIR spectroscopy. The spectra obtained were statistically analyzed by SIMCA for the recognition of regions (characteristic bands). The strongest differences obtained with the classification models were observed between 1200 and 900 cm^−1^, related to stretching vibration signals from polysaccharides in the cell wall. This technique provides the necessary information to identify and control the cheese and dairy starter cultures, finding application in the production of superior quality cheese [87].

Similarly, it is possible to identify and classify microorganisms using Raman spectroscopy. The SERS technique (with Ag substrate) has been used to identify and classify three different bacterial species (non-probiotic): *Staphylococcus aureus* and *Enterococcus faecalis* (Gram-positive) and *Pseudomonas aeruginosa* (Gram-negative). The characterization and classification of these three different species were based on spectral data using emerging clustering methods, namely fuzzy c-means and fuzzy linear discriminant analysis combined with principal component analysis (FLDA-PCA) [55]. Raman spectroscopy was also used to identify and distinguish wine lactic acid bacteria strains (*Lactobacillus*, *Pediococcus*, and *Oenococcus* strains) by comparing the signals from the components of the bacterial cells. The accurate discrimination of the *Lactobacillus* and *Pediococcus* species was accomplished by the differences in the Amide I band of proteins (1700–1600 cm^−1^) and the Amide III band (1350–1200 cm^−1^), while the symmetrical CNC stretching vibration of protein region (900–800 cm^−1^) facilitated the discrimination of *Oenococcus* as well as *Lactobacillus* and *Pediococcus*. The polysaccharides’ signatures (C–O and the C–C stretching vibrations) are found in the 1190–945 cm^−1^ region, which contributed to the accurate discrimination of *Lactobacillus* species and *Oenococcus* strains. Last, the lipid, phospholipid, and membrane signature region (CH_2_ asymmetric at ~2930 cm^−1^ and symmetric at ~2850 cm^−1^, stretching bands, C=O stretching vibration of lipid esters at 1750–1730 cm^−1^, and the CH_2_ rocking vibration at 730–715 cm^−1^) contributed substantially to the accurate discrimination of *Lactobacillus* species, while the DNA and RNA characteristic bands (PO_2_^–^ symmetric stretching at ~1090 cm^−1^ and PO_2_^–^ asymmetric stretching at ~1230 cm^−1^, as well as vibrations of the phosphate–sugar backbone of nucleic acids at 820–780 cm^−1^) facilitated the discrimination of *Oenococcus* strains as well as *Lactobacillus* and *Pediococcus* species [88].

**Figure 3:**
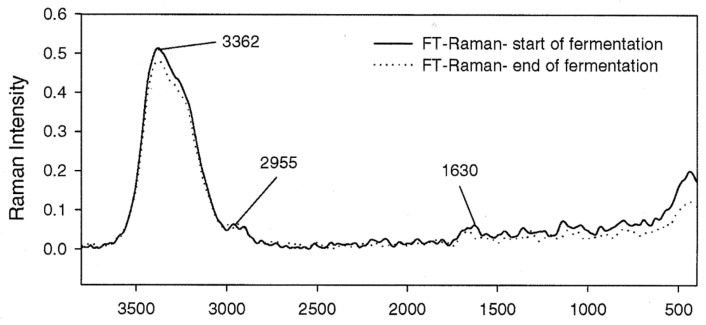
FT—Raman spectrum of *L. casei* culture at the initial and final stages of fermentation with the key band assignments. Reprinted with permission from Ref. [32]. 2001, Elsevier.

Sivakesava et al. used FT-MIR and FT-Raman to identify the structural components of *Lactobacillus casei* and the chemical components present in the culture medium at the beginning and the end of the fermentation process (Figure 3). The spectral range 1800–1000 cm^−1^ showed differences in the FT-MIR spectra due to glucose consumption and lactic acid production by *L. casei*. Correlations between spectral bands and concentrations of glucose (at 1130–950 cm^−1^), lactic acid (at 1800–1625 cm^−1^) and cell density (2950–2800 cm^−1^) were identified using calibration methods such as PCR and PLS. In the analysis of FT-Raman spectra, these bands were weaker, suggesting that the target analytes are required in higher concentrations in order to be measured by FT-Raman compared to FT-MIR [32].

**Figure 4:**
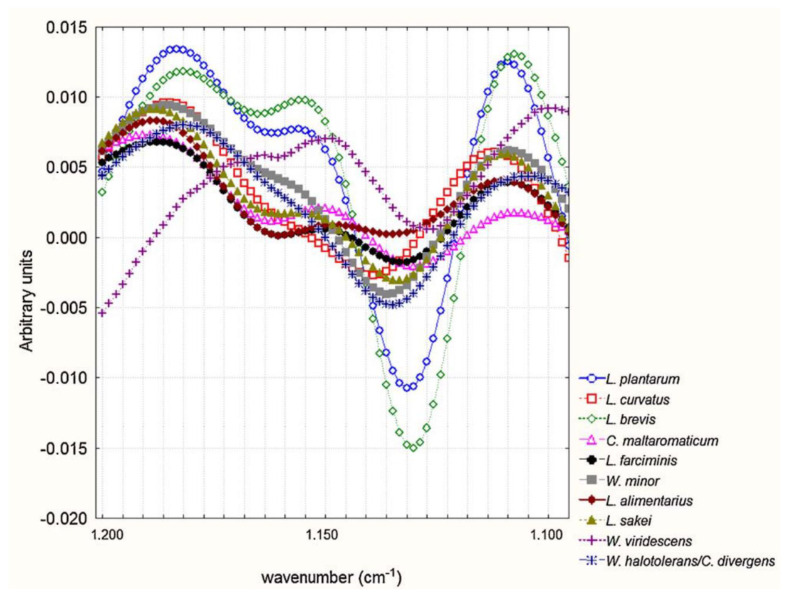
FTIR spectra of 10 classes elucidated by means of the canonical discriminant analysis, depicted in a subdivision of the 1200–900 cm^−1^ spectral windows. Reprinted with permission from Ref. [47]. 2011, Elsevier.

Mouwen et al. also made a taxonomic study of various species of lactic acid bacteria using FTIR spectroscopy, based mainly on the cell wall chemical composition differences, carrying out HCA and stepwise discriminant analysis (SDA) for the classification. The main differences between the various groups of *Lactobacillus* species appeared in the spectral ranges 1500–1200 cm^−1^, 1200–900 cm^−1^, and 900–700 cm^−1^. The spectra range between 1200 and 900 cm^−1^ is associated with their polysaccharide content, and it was defined as the most appropriate for the classification of lactic acid bacteria species (Figure 4). Three main clusters with other subclusters were also identified, based on the presence or absence of teichoic acid in their cell wall, and the amino acids composing an oligopeptide of the cell wall linkage. *Lactobacillus sakei*, *L. curvatus*, *L. farciminis*, *Carnobacterium maltaromaticum*, and *L. alimentarius* were included in the first cluster, lacking teichoic acid in their cell wall (absence of huddle between 1760 cm^−1^ and 1730 cm^−1^). Moreover, for these bacteria, the oligopeptide between the N-acetylglucosamine and N-acetyl muramic acid units consists of Lys-D-Asp, except for *C. maltaromaticum*, which is direct m-DAP (mesodiaminopimelic acid). *Weissella minor*, *W. viridescens*, *W. halotolerans* and *Carnobacterium divergens* were included in a second group, possessing teichoic acid, which is composed of either glycerol (*W. minor*, *W. halotolerans* and *Carnobacterium divergens*) or ribitol (*Weissella viridescens*), and the amino acids in the oligopeptide are Lys-Ala-Ser. Finally, the third group included *L. brevis* and *L. plantarum*, also possessing teichoic acid in their cell wall, while Lys-D-Asp (*L. brevis*) or direct m-DAP (*L. plantarum*) were the amino acids in the oligopeptide from the cell wall linkage. Although the classical grouping depends on the type of fermentation (obligatory homo-, facultative hetero-, and obligatory hetero-fermentative lactobacilli), it appears that it rather depends on the chemical composition of the cell wall of the lactic acid bacteria [47].

**Figure 5:**
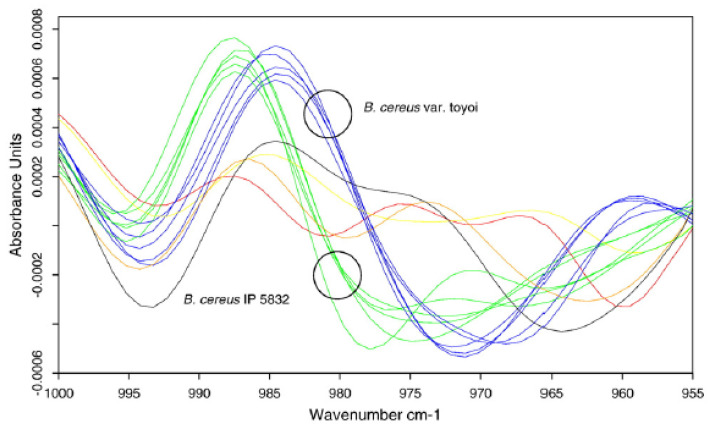
Absorption spectra (2nd derivation) with five strains, each with probiotic *B. cereus* CIP 5832 (green), *B. cereus var. toyoi* (blue), one sample of *B. cereus* type strain (red), *B. weihenstephanensis* (yellow), *B. thuringiensis* (black), and *B. mycoides* (orange), respectively, over the wave number range 1000–955 cm^−1^. Reprinted with permission from Ref. [89]. 2010, Elsevier.

Furthermore, FTIR analysis is valuable for the identification of *B. cereus* probiotic strains from pathogenic *Bacillus* bacterial strains. A hierarchical cluster analysis (HCA) was used to differentiate between these two types of strains, mainly based on the discriminatory information contained in the spectral ranges 3000–2800 cm^−1^ (fatty acid region), 1200–900 cm^−1^ (carbohydrate region), and 900–700 cm^−1^ (fingerprint region). The differences in the spectra were particularly noticeable when the second derivative was used within the wavenumbers 1000–940 cm^−1^ (Figure 5) [89].

Last, Artificial Intelligence and machine learning are playing an increasingly significant role in analyzing FTIR and Raman spectra for various aspects of bacterial analysis [76]. One prominent application is automated bacterial identification and classification. AI algorithms, including support vector machine (SVM), Partial Least Squares Discriminant Analysis (PLS-DA), Random Forest, artificial neural networks (ANNs), and convolutional neural networks (CNNs), are being used to classify bacteria at different taxonomic levels based on their unique spectral fingerprints. For instance, Ho et al. generated an extensive dataset of bacterial Raman spectra and applied deep learning approaches, achieving isolate-level accuracies exceeding 82% for identifying 30 common bacterial pathogens [79]. SERS and Raman spectroscopy have been widely applied for rapid, culture-free detection of foodborne pathogens (e.g., *E. coli*, *Salmonella*, and *Listeria*) in food matrices. Handheld Raman devices combined with chemometric models show high accuracy for monocultures but often require enrichment steps for mixed cultures, emphasizing the need for sample isolation in complex food systems [99,100,101,102,103]. The combination of Raman spectroscopy and machine learning has also shown high accuracy in identifying microbial species, with one preliminary study reporting an average classification accuracy of 95.64% using a framework of CNN and Raman spectroscopy [104]. Similarly, FTIR, NIR, and Raman spectroscopy, integrated with multivariate analysis, enable high-throughput, non-destructive metabolomic fingerprinting for food quality and safety, including discrimination of bacterial and fungal strains in complex matrices [82,105].

### 4.2. Bacterial Viability Quantification

FTIR and Raman vibrational spectroscopy can also be used to quantify and study the viability of the bacterial cells. FTIR spectroscopy, for example, was used in parallel with microbiological analysis (lactic acid bacteria (LAB), and total viable count (TVC)) to predict the growth of bacteria in pre-treated ham slices. A modeling approach was employed, where the PLS regression model provided quantitative estimations of the microbial counts, utilizing the FTIR measurements as input variables, and the counts of each microbial group as output variables. A relatively strong correlation was observed among the estimated counts by FTIR and those observed by microbiological analysis, revealing a novel and rapid computational method that could find applications in food safety [92]. Papadopoulou et al. adopted an similar approach, employing PLSR and support vector machine regression to develop classification models with satisfactory performance in rapidly estimating the microbiological counts (TVC, LAB, and lactic cocci/*S.thermophilus*) in yogurts supplemented with *Lactobacillus plantarum* T571 [72].

Similar to the previous study, Raman spectroscopy (using a portable Raman device) was used to quantify the TVC in pork samples through a designed predictive PLSR chemometric model, while the plate count method served as a reference for quantification. The predictions made for TVC concentrations below 10^7^/cm^2^ were more accurate; however, above this concentration, the PLSR model was less reliable for the exact quantitative prediction. Nevertheless, the quantification at lower concentrations is more relevant for such products, displaying the potential of the Raman scanner as a tool to detect spoilage to a given threshold, as well as quantify the TVC at a given range of concentrations [93].

FTIR and Raman methods have also been used to identify and enumerate the viability of *Staphylococcus aureus* and *Lactococcus lactis* ssp. *cremoris* bacteria in UHT milk. Multivariate statistical methods (cluster analysis, PLS, and kernel PLS) were utilized for the quantification of the bacterial species from pure cultures of *S. aureus* and cocultures with *L. cremoris.* Accurate predictions were obtained from FTIR spectra, but not from Raman spectra. A PLS2 model was then developed by combining the logarithmic viable count (log (VC)) data sets obtained for the two monocultures of *S. aureus* and *L. cremoris* to study the metabolic activity of the co-culture. Against all expectations, there was no indication of growth inhibition of *S. aureus*. Conversely, it is the growth of *L. cremoris* that would have been limited by the presence of *S. aureus*; however, the metabolic activity of the coculture was dominated by *L. cremoris*, due to its’ fermentative metabolism [85].

In the same way, Neal-McKinney et al. used FTIR and Raman spectroscopy to analyze the viability of the bacterium *Campylobacter jejuni*, a pathogen found in foods of animal origin. FTIR and Raman spectroscopies were used to determine the mechanism of inactivation of *C. jejuni* by lactobacilli, in order to predict the viability of *C. jejuni* in pure culture or co-culture with *Lactobacillus* strains. The bacterial viability was quantitatively measured from FTIR spectra using a predictive PLSR model, which demonstrated accurate predictions. By developing a hybrid PCA-PLSR model, it was also possible to analyze the metabolic activity of the co-cultured bacteria from the FTIR spectra obtained. *Lactobacillus crispatus* had a lethal effect on *C. jejuni* and dominated in co-culture, having a dominant metabolic activity, especially for lactic acid production [91].

The viability of *Lactobacillus rhamnosus GG* (LGG) probiotics encapsulated in Eudragit^®^ S100 microspheres by spray-drying was investigated using Raman-SERS by quantifying the encapsulated probiotics within the microparticles, and after incubation in gastric and intestinal fluid. More specifically, the Raman-SERS method was compared to the plate count method for quantification of probiotics, by plotting the intensity of the SERS signal at 735 cm^−1^ against the concentration of LGG from 7.6 × 10^5^ CFU/mL to 9.2 × 10^6^ CFU/mL. The signal at 735 cm^−1^ is assigned to the polysaccharides of the peptidoglycan bacterial cell wall, the adenine-containing molecules, i.e., DNA, RNA, and ATP, located on/in the cell wall, or metabolites, i.e., AMP, hypoxanthine, xanthine, and uric acid deriving from the purine degradation pathway in the extracellular regions near the outer cell walls [90].

### 4.3. Bacterial Stability Assessment

Evaporation of water by freeze-drying and spray-drying is used to extend probiotics’ viability, and relatively low molecular weight carbohydrates have been utilized as effective protective agents during such harsh conditions [13]. One hypothesis is that the carbohydrates bind to the polar heads of lipid membranes by hydrogen bonds, taking the place of the removed water. The carbohydrate molecules interact with phosphates through hydrogen bonds. In the absence of the protective agent and water, a shift in the vibrational mode of asymmetric stretching of the phosphate (~1230 cm^−1^) towards higher wavenumbers is observed [106]. However, in the presence of the carbohydrate (without water), this shift is not observed. The band is observed at the same wavenumber as hydrated membranes [106,107]. The protective capacity of carbohydrates by replacing water in the lipid membranes was not observed using higher molecular weight molecules, such as maltodextrins or hydroxyethyl starch, despite the fact that these molecules can facilitate the interactions presented [13]. The protective capability of sugars was evidenced by the enhanced survival of encapsulated *Lactobacillus rhamnosus GG* (LGG) probiotics during exposure to simulated gastric and intestinal fluids. Carbohydrates with small molecular size, such as mannitol and trehalose, were more effective in their protective capacity, as they are able to interact directly with the polar heads of membrane phospholipids [90].

Another hypothesis suggests that carbohydrates allow the probiotics to remain in the amorphous state, in which molecular interactions are restricted, and then prevent the glassy transition to a less stable rubbery state, where the biochemical reactions can be detrimental [108]. Using FTIR, the glass transition (Tg) can be determined by studying the strength of the hydrogen bonds. Weak hydrogen bonds between the carbohydrates and water are characteristic of an amorphous state of the matrix, while strong hydrogen bonds are linked to a rubbery state [13]. A glass transition from the amorphous to the rubbery state leads to a significant shift in the vibrational stretching mode of -OH towards lower wavenumbers in the spectrum [109,110]. Both hypotheses involve the formation or destruction of hydrogen bonds, which can be observed using FTIR spectroscopy [13].

**Figure 6:**
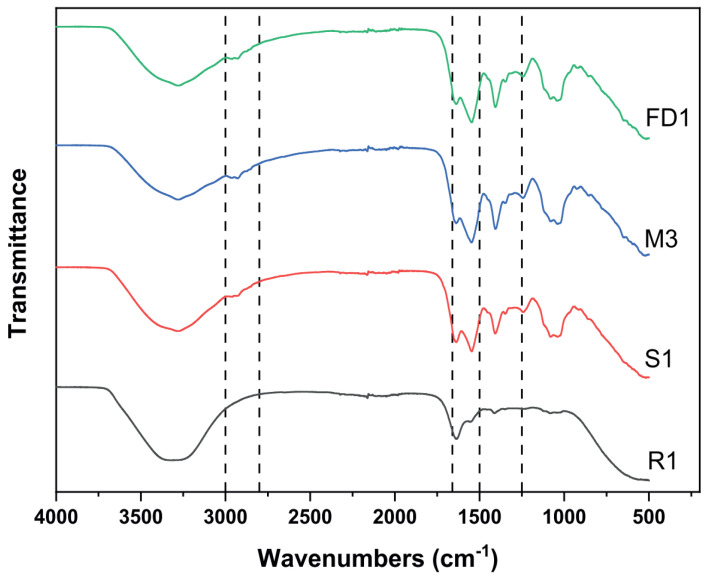
FTIR spectra of Bifido probiotic cells after 2 h of EHD (samples S1 and M3), as well as the freeze-dried (sample FD1) and the non-dried sample (reference sample R1). Figure reproduced from [94], with y-axis values presented in arbitrary units (a.u).

FTIR spectroscopy has been utilized as a method to assess the drying process of probiotics. For example it has been applied to investigate the electrohydrodynamic drying (EHD) of probiotics using ionic wind [94]. The EHD process was found to be dependent on several parameters, and the FTIR technique proved effective in revealing differences between various samples. Figure 6 illustrates the spectra of *Bifidobacterium animalis* subsp. *lactis* (Bifido) probiotic cells, highlighting the main characteristic bands of the probiotic cells both in suspension (R1) and after drying. Notably, there is a band around 3280 cm^−1^, indicative of -OH stretching. This band is more prominent in the non-dried probiotics due to their higher water content. On the contrary, the fatty-acid region, ranging from 3000 to 2800 cm^−1^, as well as the bands at 2966, 2936, and 2876 cm^−1^ corresponding to the asymmetric stretching of CH_3_, the asymmetric stretching of CH_2_, and the symmetric stretching of CH_3_ within the nonpolar region of the phospholipid bilayer, respectively, were only visible in the dried samples (S1, M3, and FD1). The FTIR spectra in Figure 6 also display bands related to membrane proteins of probiotics, specifically the carbonyl stretching of secondary amides (Amide I) at 1639 cm^−1^ and the N–H bending of Amide II at 1545 cm^−1^. The Amide I absorption band was more intense in samples with higher water content, whereas the intensity of the Amide II band was associated with water loss, being more prominent in the more thoroughly dried samples (FD1 and M3). Phosphate vibrations (PO_2_) at 1250 cm^−1^, and carbohydrate and phosphate bands between 1100 and 980 cm^−1^ were observed in all samples. Meanwhile, the bands at 1070 cm^−1^ and 1040 cm^−1^, which correspond to C–O–C vibrations of the polar site of the phospholipid bilayers, showed a decrease in intensity in the non-dried samples [94].

FTIR and Raman spectroscopy have been proven effective for investigating interactions between *Bifidobacterium animalis* subsp. *lactis* (Bifido) and lecithin phospholipids, and ultimately for assessing their protective effects on probiotics’ viability [95]. FTIR and Raman spectroscopy indicated that Bifido cells interacted with lecithin molecules predominantly through hydrophobic interactions. Those interactions could be controlled by the concentration of lecithin. As the concentration of lecithin increased, a gradual shift was observed in the fatty acid spectral range (3100 to 2800 cm^−1^). In FTIR, peaks attributed to Bifido at 2960 and 2927 cm^−1^ faded and were progressively overlaid by prominent lecithin peaks at 3011, 2955, 2924, and 2853 cm^−1^. This suggests interactions between the –CH groups in the bacterial cell lipid wall and lecithin. Raman spectra of Bifido-lecithin samples revealed two significant peak shifts from the spectrum of pure Bifido. These shifts included a change in the C=O stretching vibration band from amide groups, moving from 1667 to 1656 cm^−1^, and a shift in the CH_2_ deformation band from 1457 to 1446 cm^−1^. Similar to FTIR spectra, Raman spectra also showed Bifido peaks shifting closer to the characteristic peaks of lecithin, with peak intensities increasing in line with higher lecithin concentrations. However, the persistence of the peak at 2937 cm^−1^ from the Bifido spectrum in the Bifido-lecithin samples, without any shift in position, suggested that the observed spectral changes are due to the interactions between these components [95].

Overall, the shifts identified in both FTIR and Raman spectra mainly occur for the same functional groups, such as amide and alkane (CH_2_) groups, indicating interactions between lecithin and the Bifido probiotic membrane, particularly in specific regions of both components. The shift in CH_2_ groups suggests interactions within the hydrophobic regions of the Bifido probiotic membrane and lecithin, which is further supported by the overlapping of Bifido peaks with lecithin peaks in both FTIR and Raman spectra, especially as lecithin concentration increases. Additionally, the Bifido probiotic strain is known to be highly hydrophobic, potentially facilitating hydrophobic interactions with the hydrophobic regions of lecithin, such as the alkyl chains. Shifts in amide groups point to potential interactions with Bifido membrane proteins, a phenomenon observed in several studies, which suggest that added phospholipid ingredients (such as milk phospholipids) interact with bacterial cell surface proteins Finally, additional shifts observed in FTIR spectra for P-O-C and P=O groups imply that interactions may occur with phosphoric groups in bacterial lipid structures (such as phospholipids), as well as bacterial cell lipopolysaccharides [95].

Furthermore, FTIR has been used to confirm the encapsulation of probiotics. For instance, *Bifidobacterium animalis* subsp. *lactis* probiotics encapsulated within electrosprayed ethyl cellulose core–shell microcapsules were not detected at the surface of the capsules, suggesting suitable encapsulation of the bacterial cells within the microcapsules [49]. Higher levels of entrapment may lead to greater probiotic stability.

In another study, the encapsulation and drying of *Bifidobacterium animalis* subsp. *lactis* (Bifido) cells in maltodextrin microcapsules using electrospray processing were investigated [96]. FTIR was used to demonstrate the influence of an external electric field by electrospraying at positive and negative polarity. Notable differences were observed in band intensity within the fatty-acid region, ranging from 3000 to 2800 cm^−1^, with the electrosprayed samples exhibiting more pronounced bands compared to the non-treated Bifido. Similarly, the bands at 2961, 2930, and 2876 cm^−1^, which are attributed to the asymmetric stretching of CH_3_, CH_2_, and the symmetric stretching of CH_3_, respectively, within the nonpolar site of the phospholipid bilayer, were also more intense in the electrosprayed samples. More specifically, the Bifido samples electrosprayed with negative polarity showed higher transmittance intensity compared to those electrosprayed with positive polarity. Consequently, the intensity of the peaks for the –CH_3_ and –CH_2_ groups correlated with the water content in the samples, decreasing in the sequence: negative polarity > positive polarity > no electric fields, where these peaks are not visible due to the highest water content. The differences in the water content of the samples were confirmed by the protein amide bands. As mentioned earlier, the Amide I absorption band is more intense in samples with higher water content, while the Amide II absorption is notable in the samples with lower water content. The carbonyl stretching vibrations from secondary amides (around 1640 cm−1, Amide I) were more prominent compared to the N–H bending and C–N stretching vibrations of the Amide II peak (around 1550 cm^−1^) in the non-treated Bifido. However, for the electrosprayed Bifido, a less pronounced Amide I peak relative to Amide II was observed, with the least intensity for the sample electrosprayed using negative polarity. Additionally, the intensity changes in the polar regions of cell phospholipid bilayers at 1070 cm^−1^ and 1040 cm^−1^ (from C–O–C vibrations) reveal that less pronounced peaks in non-electrosprayed samples suggest more water interaction, while stronger peaks in negatively electrosprayed samples indicate reduced water content compared to positively electrosprayed ones [96].

### 4.4. Bioprocess Monitoring

Vibrational spectroscopic methods can also be used to monitor bioprocesses in real time. Monitoring the lactic acid production by *Lactobacillus casei* (*L. casei*), by FTIR spectroscopy, combined with the use of PLS and the HPLC as a reference method, allowed the measurement of lactose, galactose, lactic acid, and biomass concentrations during lactic fermentation [98,111]. Suitable spectral wavenumber regions from FT-MIR, NIR and FT-Raman were chosen to develop calibration models and were used to monitor biomass, glucose, and lactic acid production by *L. casei*, allowing rapid bioprocess optimization [32]. In another study, FTIR spectroscopy was used in combination with other conventional chemical methods to monitor the process of the fermentation of inoculated and wet-cooked sorghum flour by measuring the increase in titratable acidity, total protein and free amino acid content, and the decrease in reducing sugars, soluble proteins, and starch content. The nutritional characteristics of sorghum were improved after fermentation by lactic acid bacteria, while the FTIR offered rapid and comprehensive information about the process [112]. In other studies, FTIR spectroscopy was used to analyze the structure and the physicochemical properties of molecules produced by probiotics, such as exopolysaccharides from *Bacillus licheniformis* AG-06 [97].

Furthermore, FTIR spectroscopy enables rapid and accurate strain typing for food safety and quality control, delivering results consistent with whole-genome sequencing. This approach is particularly effective for early screening and source tracking in food production environments [113]. Similarly, Raman microspectroscopy demonstrates strong discriminatory power for food-relevant bacteria at genus, species, and strain levels, even under stress conditions, achieving classification rates up to 97.6% [114]. In another study, SERS, enhanced with nanoparticles, enables single-cell analysis of bacterial metabolic activity in food matrices (e.g., *E. coli* in chicken carcass wash water), supporting real-time monitoring of disinfection and metabolic states [82].

## 5. Advantages and Disadvantages of Using FTIR and Raman Spectroscopies for Probiotic Analysis

FTIR and Raman methods provide reliable qualitative and quantitative information about a sample, based solely on the analysis of the functional groups of the molecules it contains [26,115]. They are powerful tools to investigate the biochemical composition of bacteria and are sensitive to even minor alterations in composition, enabling the whole organism’s fingerprinting [116,117]. That is why both methods have been applied as whole-organism fingerprinting tools: to study the effects of antimicrobial agents in bacteria, and for classification and identification of microorganisms and biofilms [118]. Other advantages of the vibrational spectroscopic methods over other conventional methods are that they are fast, non-invasive, staining- and labeling-free, and less susceptible to human subjective analysis [63]. Moreover, they require small quantities of samples as well as easy sample preparation, while being simple to use with a low cost of running per sample and an overall life cycle, without requiring frequent maintenance [64]. Cell lysis is not necessary for the analysis, being a non-invasive technology in the case of FTIR and providing a culture-free identification in the case of Raman. In addition, they are high-throughput technologies providing high sensitivity and data reproducibility, while the size and complexity of the obtained data are low [39,64,118]. Reproducibility, high performance, and quality are the key prerequisites in industrial manufacturing probiotic processes, where FTIR and Raman can be used from benchtop experiments to large-scale industrial production of probiotic strains [119]

However, both methods can also present some limitations for the analysis of biomolecules. The complexity of microbial samples could lead to overlapping FTIR spectral signals, which is one of the main drawbacks of FTIR. However, this could be alleviated by employing advanced single-cell FTIR spectroscopy. Furthermore, the inadequacy of standardized protocols and extensive databases, especially for less studied or newly discovered microorganisms, hinders the consistent and reliable microbial identification [120]. The dependency on reference analytics is also one of the main challenges of Raman spectroscopy, as most reference databases are specific to different conditions, such as the detection method, technical parameters, state of analyte, and sample preparation. For that reason, algorithms and models should be developed for the standardization of data analysis to establish comparability of results and acquire a validated method for industrial applications [39].

Moreover, IR absorption is sensitive to changes in the intrinsic dipole moment that occur with molecular vibrations. Polar groups, such as C=O, N–H and O–H, thus exhibit strong IR stretching vibrations. Water is a polar molecule that exhibits very strong IR absorption, which will then create unwanted interference on the resulting spectrum. To counteract this effect, an ATR accessory can be used [26]. The presence of water does not prominently affect the collection and analysis of a Raman spectrum. In addition, Raman spectroscopy provides more spectral features than FTIR spectroscopy and often offers higher specificity of analysis with narrower spectral bands [52,56]. In addition, the lowest FTIR detection limit of bacterial cell concentration is 10^3^ CFU/mL, which can be improved by the utilization of a membrane filter matrix in combination to concentrate the bacterial cells, and with the use of relatively pure cultures and controlled culture conditions. Raman spectroscopy, on the other hand, is differentiated from FTIR spectroscopy by its cultivation independence for the analysis of the bacterial cells [73].

However, FTIR spectroscopy is simpler to use than Raman. Moreover, the spectral signals from conventional Raman spectroscopy are weak, but the use of the SERS technique can enhance the signals obtained by several orders of magnitude. Other limitations of Raman technology include the damage to the sample that can occur due to the intense laser powers typically used, especially when using the visible wavelength range, which can burn and alter the sample under study [56]. Thus, considering all of these elements, it is preferable to use FTIR and Raman spectroscopies in a complementary way, as they both have advantages for the analysis of probiotics and other types of bacteria, and can offer a more comprehensive approach to the analysis of probiotic samples and ensure more detailed chemical information [26,63].

Furthermore, other limitations and challenges of using those techniques in bacterial analysis in food systems can include: (i) Matrix interference: complex food matrices often interfere with spectral signals, making efficient sample preparation and matrix-matched calibration essential for reliable results [82,101,103]. (ii) Sensitivity and reproducibility: while SERS provides high sensitivity, it can suffer from variability due to substrate preparation and sample handling. FTIR and Raman generally offer lower sensitivity for trace-level detection compared to molecular methods [82,121]. (iii) Standardization and data interpretation: the absence of universal spectral databases and inconsistencies in data processing hinder cross-study comparability and routine implementation. Advanced chemometric and machine learning tools are improving interpretation but require further standardization [101,105]. (iv) Single-cell analysis: Raman and SERS can achieve single-cell detection, but technical challenges such as thermal damage, spectral artifacts, and low signal-to-noise ratios limit their widespread use in food microbiology [122,123]. (v) Throughput and cost: although FTIR and Raman are rapid and cost-effective compared to culture-based methods, large-scale implementation in food industry settings faces challenges related to instrument cost, automation, and workflow integration [100,105].

## 6. Conclusions and Perspectives

The need to identify, quantify, and characterize probiotics and other types of bacteria has increased significantly with the discovery of their powerful effect on human health. With this increasing need, the use of rapid and accurate alternative methods has intensified. Thus, the use of FTIR and Raman spectroscopies in bacteria research has been widely used.

FTIR and Raman vibrational spectroscopy methods allow real-time and *in situ* analysis of several matrix components in a single spectrum using a small amount of sample. Different FTIR techniques (such as ATR and specular reflection) and different Raman techniques (such as tip-enhanced TERS and surface-enhanced SERS) have been developed, while the chemometric methods have also been used to develop various calibration models, allowing a complete analysis of the structure and composition of the samples.

Both spectroscopies can be utilized to study a large diversity of probiotic samples and bacteria for the identification of their various taxonomic groups, as well as for quantification and determination of their viability. Other applications of FTIR and Raman analysis include monitoring of bioprocesses to determine various compounds in real time, e.g., during fermentation of probiotics, and evaluation of the effect of protectants and encapsulating agents on the long-term stability and viability of probiotics after freeze drying, spray drying, and electrospray processing technologies.

While both FTIR and Raman spectroscopy have their respective limitations, e.g., FTIR’s challenges with aqueous samples and Raman’s susceptibility to fluorescence interference, ongoing technological advancements and dedicated research efforts are continuously addressing these issues. Notably, the increasing availability of affordable, portable, and handheld spectrometers is opening new possibilities for real-time, on-site analysis in industrial settings. These tools are becoming more viable for applications in the probiotics industry, particularly when combined with innovations such as Artificial Intelligence (AI), advanced fluorescence mitigation techniques, and the development of comprehensive spectral libraries. These advancements are expected to yield more accurate, sensitive, and user-friendly portable systems that can complement or, in some cases, replace traditional benchtop instruments. Ultimately, this progress supports more robust and efficient quality control processes, particularly within probiotics and bacterial analysis in the food industry. However, it is crucial to address the existing challenges related to matrix interference, reproducibility, and standardization, which must be addressed for broader adoption in food safety and quality assurance. Challenges on data quality, model interpretability, ethical considerations, and regulatory pathways should also be considered to ensure the widespread adoption and successful translation of AI-powered spectroscopic methods in bacterial analysis. Future developments in NIR and Raman spectroscopy for bacterial analysis are expected to increasingly integrate AI-driven chemometric models with Internet of Things (IoT) connectivity, enabling real-time analysis, on-site monitoring, remote data transmission, and automated decision-making in food safety systems.

Overall, the ability of these two spectroscopies to deliver non-invasive, rapid, and detailed insights into the molecular composition and structure of probiotics and bacteria opens promising avenues for diverse applications, while also paving the way for further exploration and innovation. Together, these techniques offer a powerful approach to advancing probiotics and bacteria research by enabling scientists to fine-tune bacterial strains for specific attributes and improving product development and quality assurance across the food and biotechnology industries.

## Figures and Tables

**Table 1:** FTIR and Raman spectra main bands assigned to the different components of bacterial samples [63,64].

FTIR Frequency (cm^−1^)	Assignment	Raman Frequency (cm^−1^)	Assignment
**3350−3200**	ν (O–H), ν (N–H), water, Amide A	**~** **3064**	ν (=C–H), proteins, lipids
**2960−2950**	$v_{as}$ (CH_3_), lipids	**3100−2800**	ν (CH_3_), ν (CH_2_), lipids, carbohydrates, proteins
**2940−2920**	$v_{as}$ (CH_2_), lipids		
**2860−2850**	$v_{s}$ (CH_2_), lipids		
**1740−1730**	ν (C=O), phospholipids	**1700−1600**	ν (C=O), Amide I
**1700−1600**	80% ν (C=O), 20% ν (C–N), τ (HOH), Amide I, water	**1667−1650**	ν (C=C), lipids, proteins
**1600−1500**	60% τ (N–H), 30% ν (C–N), 10% ν (C–C), Amide II	**1600−1500**	ν (C–N), δ (N–H), Amide II
		**1576**	adenine, guanine (DNA bases)
		**1523**	cytosine (DNA bases)
		**1500−1400**	in-plane τ and out-of-plane τ (CH_2_), lipids
**1462−1441**	Pyrrolidine ring vibration of proline and hydroxyproline	**1461−1445**	$v_{s}$ (CH_2_), saturated lipids
**1450−1400**	$\delta_{as}$ (CH_3_), $\delta_{as}$ (CH_2_), proteins, lipids		
**1400−1350**	$\delta_{s}$ (CH_3_), $\delta_{s}$ (CH_2_), $v_{s}\;$(C=O), proteins, lipids	**~** **1380**	δ (COH), (HCO), (HCC), $v_{s}$ (COO–), (C–O), polyanionic polysaccharide
**1350−1200**	τ (N–H), ν (C–N), τ (C=O), ν (C–C), ν (CH_3_), Amide III	**1340−1330**	Polynucleotide chains, DNA purine bases
		**1330−1125**	Trans ν (C–C), lipids
		**1300−1250**	In-plane τ and out-of-plane τ (CH_3_), lipids
		**~** **1280**	δ (COH), (HCO), (HCC), $v_{s}$ (COO–), (C–O), polyanionic polysaccharide
		**1300−1230**	ν (C–N), δ (N–H), Amide III
**1242−1230**	$v_{as}$ (PO_2_^–^), DNA, RNA, phospholipids, phosphorylated proteins	~**1260 (shoulder band)**	δ (CH), lipids, proteins
		**1200−1050**	ν (C–C), lipids
**1144−1137**	Oligosaccharydes	**1075, 1055, 980–880**	Combination of rhamnose, galactose, and glucose
		**1127**	ν (C–N), proline
		**1125**	glucose
		**1120**	$v_{s}$ (COC), glycosidic bonds
		**1094**	$v_{as}$ (COC), (1→4)-β-linked glycosidic bonds
**~1086**	$v_{s}$ (PO_2_^–^), DNA, RNA, phospholipids, phosphorylated proteins	**~1068**	Trans ν (C–C), lipids
**1080−1070**	ν (C–C), β−glucan bonds	**1000**	Phenylalanine ring breathing
**1046−999**	Skeletal vibration connected to anomeric structure of D−glucose		
**1016−1009**	ν (C–C), RNA, ribose		
**~972**	ν (C–C), ν (C–O), DNA, deoxirobose	**950−790**	Side group δ (COH), (C–CH), (O–CH), carbohydrates
**900−700**	Anomeric ring vibrations for tryptophan, tyrosine, phenyloalanine		
**929**	(1→3)−α−D−glucan	~**948**	(1→3)-α-D-glucan
**860−852**	(1→3),(1→6)−α−D−glucan	**800−640**	Out-of-plane τ (N–H), Amide V
		**852**	(1→6)-α-D-glucan
		**~783**	Ring breathing of cytosine, thymine, uracil; $v_{s}$ (O–P–O), phosphodiester bonds in DNA
		**770–625**	τ (O=C−N), Amide IV
		**~757, ~520**	Glucans
		**600−540**	Out−of−plane τ (C=O), Amide VI
		**380**	β−D−glucoside

Types of vibrations: stretching (ν), deformational (δ), bending (τ), symmetrical (s), and asymmetrical (as) modes.

**Table 2:** Overview of some IR and Raman spectroscopy applications in bacterial analysis.

Application Area	Bacterial Strain	Spectroscopy Type	Information Collected	Reference
**Taxonomic Analysis and Bacterial Identification**	*Staphylococcus aureus*, *Lactococcus lactis* ssp. *cremoris*	FTIR and Raman	Identification, quantification and viability/dominant metabolism in coculture.	[85]
	*Lactobacillus alimentarius*, *L. brevis*, *L. curvatus*, *L. farciminis*, *L. plantarum*, *L. sakei*, *Carnobacterium divergens*, *C. maltaromaticum*, *Weissella halotolerans*, *W. minor*, and *W. viridescens*	FTIR	Taxonomic classification of lactic acid bacteria from the genera *Lactobacillus*, *Weissella* and *Carnobacterium.*	[47]
	*Staphylococcus aureus*, *Enterococcus faecalis*, and *Pseudomonas aeruginosa*	Raman SERS	Identification and classification of three different bacterial species.	[86]
	*Lactobacillus* spp, *Streptococcus thermophilus*, and *Propionibacterium* *freudenreichii*	FTIR-ATR combined with hydrophobic gridded membrane filters	Classification of *Lactobacillus* culture at a strain level (to be able to select a culture at a strain level).	[87]
	*Lactobacillus*, *Pediococcus* and *Oenococcus strains*	Raman	Identification and discrimination of wine lactic acid bacteria.	[88]
	Strains of *Bifidobacterium* genera	FTIR	Isolation of *Bifidobacteria* from food and human feces and identification.	[50]
	*Bacillus cereus*	FTIR	Separate probiotic strains from wild-type bacteria/distinguish pathogenic from non-pathogenic *B. cereus.*	[89]
**Bacterial Viability Quantification**	*Lactobacillus rhamnosus GG*	Raman SERS	Quantification and viability of micro-encapsulated probiotics/Protection ability of carbohydrates for probiotics	[90]
	*Campylobacter jejuni*, *Lactobacillus crispatus*, *L. gallinarum*, *L. helveticus*, and *L. acidophilus*	FTIR and Raman	Prediction of viability and investigation of competitive exclusion of *Campylobacter jejuni* by Lactobacilli (production of lactic acid).	[91]
	Lactic acid bacteria (LAB) and total viable counts (TVC)	FTIR	Quantification of LAB and TVC and characterization of the state of spoilage of ham slices.	[92]
	TVC	Raman	Quantification of TVC and characterization of the state of spoilage of pork.	[93]
**Bacterial Stability Assessment**	*Bifidobacterium animalis* subsp. *lactis*	FTIR	Effect of electric fields on the extent of drying of probiotics.	[94]
	*Bifidobacterium animalis* subsp. *lactis*	FTIR and Raman	Interactions of probiotics with bacteria.	[95]
	*Bifidobacterium animalis* subsp. *lactis*	FTIR	Encapsulation of probiotics and effect of electric fields on non-encapsulated probiotics.	[49,96]
	Mix of *S. thermophilus* and *Lactobacillus delbrueckii* subsp. *Bulgaricus*	FTIR	Proper integration of the probiotic in its encapsulation matrix (alginate and chitosan matrices).	[48]
**Monitoring bioprocesses**	*Bacillus licheniformis*	FTIR	Structure and physicochemical properties of exopolysaccharides produced by *B. licheniformis.*	[97]
	*Lactobacillus casei*	FTIR and Raman	Monitor biomass, glucose, and lactic acid production by *L. casei*, allowing rapid bioprocess optimization.	[32]
	*Lactobacillus casei*	FTIR	Effects of lactic acid bacteria on MIR spectra/monitoring of lactic fermentation.	[98]

## Data Availability

No new data were created or analyzed in this study. Data sharing is not applicable to this article.
